# Post-abortion contraceptive uptake among women seeking care for incomplete or induced abortions in Northern Ethiopia: a facility-based cross-sectional study in a humanitarian setting

**DOI:** 10.3389/fpubh.2026.1911824

**Published:** 2026-09-18

**Authors:** Nakachew Sewnet Amare, Tewodros Seyoum, Amare Tariku, Cristina Mattison, Marie Klingberg-Allvin

**Affiliations:** 1 Department of Women and Family Health, School of Midwifery, College of Medicine and Health Sciences, University of Gondar, Gondar, Ethiopia; 2 School of Nursing and Midwifery, Asrat Woldeyes Health Science Campus, Debre Berhan University, Debre Berhan, Ethiopia; 3 Department of Women’s and Children’s Health, Karolinska Institutet, Stockholm, Sweden; 4 Global Women's and Newborn's Health, Burnet Institute, Melbourne, VIC, Australia; 5 Department of Human Nutrition, Institute of Public Health, University of Gondar, Gondar, Ethiopia

**Keywords:** contraceptive uptake, family planning, humanitarian settings, northern Ethiopia, post-abortion care

## Abstract

**Introduction:**

Unintended pregnancy after abortion is a major concern in humanitarian settings, where access to contraceptive services is often limited. Increasing access to post-abortion contraceptives is especially vital for women and girls in these contexts. However, evidence from conflict-affected Ethiopia remains limited. This study aimed to assess post-abortion contraceptive uptake and its associated factors among women who received care for incomplete or induced abortions in humanitarian settings in the Amhara region of Ethiopia.

**Methods:**

A facility-based cross-sectional study was conducted among 1,642 participants at 31 selected public health facilities across seven humanitarian zones affected by the northern Ethiopian conflict in the Amhara Region. A semi-structured questionnaire was used to collect the data. A binary logistic regression model was used to identify factors significantly associated with the outcome; adjusted odds ratios were reported for *p*-values <0.05, along with 95% confidence intervals.

**Result:**

The overall post-abortion contraceptive uptake in the study setting was 27.8% [95% CI: 25.6–30%]. The odds of post-abortion contraceptive uptake were significantly higher among women with single marital status [AOR: 1.96; 95% CI: 1.39–2.75], a previous history of induced abortion [AOR: 2.00; 95% CI: 1.42–2.83], and those who received a high level of person-centered comprehensive abortion care (PC-CAC) services [AOR: 3.39; 95% CI: 2.39–4.82]. In contrast, the odds were significantly lower among women and girls with a history of spontaneous abortion [AOR: 0.73; 95% CI: 0.55–0.97].

**Conclusion:**

Post-abortion contraceptive uptake in this humanitarian setting was low, indicating a substantial gap in the provision of post-abortion care. Low uptake of contraception following abortion may increase the risk of repeat unintended pregnancies, with added consequences for women and the health system. Addressing this gap requires strengthening the delivery of high-level PC-CAC, including timely, accurate, and accessible contraceptive counseling and services for all women.

## Introduction

Humanitarian crises intensify barriers to sexual and reproductive health (SRH) services, increasing women and girls’ vulnerability to unintended pregnancies and unsafe abortions (1). In these settings, comprehensive abortion care (CAC), which includes the provision of safe induced abortion, the management of abortion-related complications through post-abortion care (PAC), and ensuring access to voluntary contraceptive services, is essential (2). These services are critical for preventing repeat unintended pregnancies and reducing maternal morbidity and mortality (3–5). Contraceptive counselling is a key component of CAC, and early initiation of contraceptives following either induced or spontaneous abortion is therefore essential for women who wish to delay or avoid future pregnancies (2, 6). The approach that ensures quality CAC and improves post-abortion contraceptive uptake by actively engaging women and girls in decision-making about their care, respecting their values and preferences, fostering effective communication, and providing individualized support throughout the process is known as person-centered comprehensive abortion care (PC-CAC) (2, 7, 8).

Evidence from humanitarian settings demonstrates substantial variation in post-abortion contraceptive uptake. For example, a study conducted in a South African hospital reported an overall uptake rate of 74.5%, which increased to more than 90% during the COVID-19 pandemic (9). A recent WHO assessment of PAC in humanitarian settings found wide variation: contraceptive counselling reached 99% of women in a CAR hospital versus 39% in Nigeria, while method uptake was 63.4% versus 4.7%, respectively (8).

Post-abortion contraceptive use is further shaped by a range of sociodemographic and contextual factors, including age, marital status, prior contraceptive use, and the quality of counseling received (10, 11). Persistent social and systemic barriers, such as poverty and limited educational opportunities, also constrain access to and uptake of post-abortion contraceptives (3, 11).

Evidence from diverse settings further reinforces the value of individualized care. For example, studies conducted in Italy and the US highlight that a person-centered approach is a key to improving women’s uptake of effective contraception (12). Similarly, evidence from Kenya indicates that PC-CAC is central to women’s satisfaction and post-abortion contraception uptake and use (13). In contrast, research among Congolese refugees in Uganda shows that legal restrictions and abortion-related stigma limit access to post-abortion contraceptive services (14). Addressing these multifaceted challenges is therefore critical to ensuring that post-abortion contraceptive services in humanitarian settings are both effective and accessible, ultimately improving reproductive health outcomes and disrupting cycles of unsafe abortion (3, 4, 15, 16).

Despite this growing body of evidence, data on post-abortion contraceptive uptake in humanitarian settings, particularly in Ethiopia, remain limited. To address this gap, the present study aims to assess post-abortion contraceptive uptake and associated factors in humanitarian settings in the Amhara region of Ethiopia.

## Materials and methods

### Study design and setting

We conducted a facility-based cross-sectional study from September 2023 to March 2024 in the humanitarian setting of the Amhara region, which was heavily affected by the conflict in northern Ethiopia. The study was conducted in public health facilities providing CAC, including referral hospitals, primary hospitals, general hospitals, and health centers, and focused on women receiving CAC services. In Ethiopia, post-abortion contraception is integrated across all levels of the health system, including the mobile health clinics in internally displaced persons (IDP) camps. Primary health care units and mobile clinics typically provide short- and long-acting methods, while secondary and tertiary facilities offer a broader range of services, including permanent methods (17). These services are delivered by a diverse group of health professionals, including midwives, nurses, level IV health extension workers, health officers, integrated emergency surgical officers (IESO), and physicians (general practitioners and specialists) (18).

### Study facilities

Thirty-one health facilities were selected from seven humanitarian zones affected by the northern Ethiopian conflict. The facilities were first stratified by type: referral hospitals, general hospitals, primary hospitals, and health centers. Before facility selection, a facility assessment was conducted with the local research team to evaluate service availability and client flow. Facilities that were not offering abortion services or had very low caseloads, making it unfeasible to reach the minimum sample size within the data collection period, were excluded from the sampling frame. Based on these criteria, we identified 119 facilities, including 3 referral hospitals, 46 primary and general hospitals, and 70 health centers. From this pool, all three referral hospitals were included, along with 14 (30%) of the primary and general hospitals and 14 (20%) of the health centers, yielding a total of 31 facilities. Given the ongoing conflict and associated security risks, four initially selected facilities were replaced to ensure the safety of data collectors and the continuity of field activities. Despite these substitutions, the total number of facilities remained consistent at 31.

### Study population

The study population comprised women and girls who received abortion care services for incomplete or induced abortions at the selected facilities during the data collection period, from September 21, 2023, to March 30, 2024, with a total of 1,642 participants, as shown in the flowchart (Figure 1).

**Figure 1:**
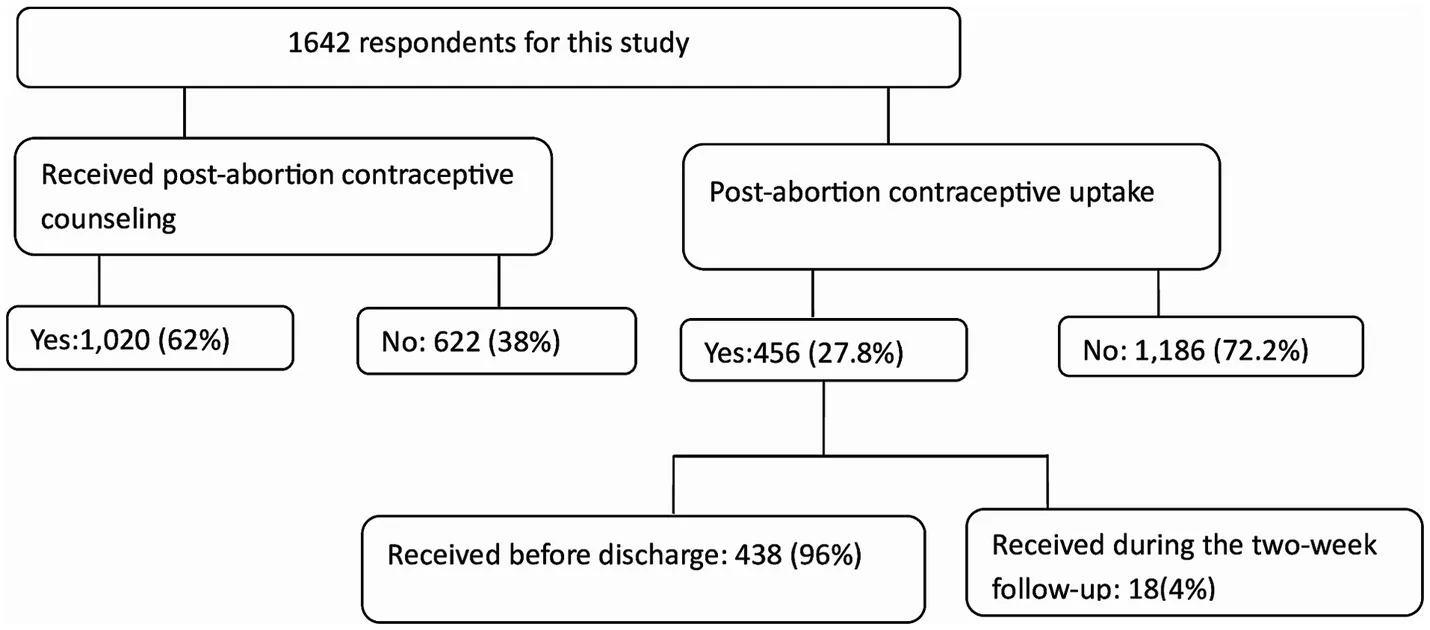
The flowchart of the study.

### Inclusion criteria

Women and girls who received abortion care services for incomplete or induced abortion at the selected study facilities during the data collection period were eligible for inclusion.

### Sample size and sampling procedures

The data for this analysis were drawn from the main study, for which the minimum required sample size was calculated to be 1,509 participants. To select the study participants, a consecutive sampling technique was employed across 31 health facilities. The estimated study population was closest to the calculated sample size, and all eligible clients presenting to the selected health facilities during the study period were consecutively enrolled. During the data collection period, a total of 1,642 eligible participants were included in the study (19).

### Data collection and quality control

The exit interviews were conducted with all participants before discharge following abortion care, alongside the assessment of PC-CAC. During the exit interview, participants who had received a post-abortion contraceptive method before discharge did not require further follow-up to assess contraceptive initiation, whereas participants who had not received a post-abortion contraceptive method before discharge were enrolled in a two-week follow-up to determine whether they subsequently initiated contraception. To facilitate follow-up, the contact information for the participants and, with participants’ permission, the contact information of their partners or family members were recorded before discharge. At the end of the follow-up period, they were contacted to determine whether they had a post-abortion contraceptive uptake.

To evaluate the person-centeredness of the care, we used the PC-CAC measurement tool, which includes comprehensive items to assess women’s perception of PC-CAC following abortion care and before discharge from the facility. The tool was initially validated in Kenya, adapted to the Ethiopian context, and content-validated by experts. The final tool, which includes variables related to sociodemographic characteristics, reproductive health history, health facility-related characteristics, abortion-related characteristics, and post-abortion contraception uptake and counselling, was initially developed in English, then translated into the local language (Amharic), and back-translated into English to ensure accuracy and consistency.

Data were collected using a semi-structured questionnaire administered via both the KoboCollect mobile application and a hard-copy version. This dual approach minimized the risk of data loss, such as from device failure. After completing the digital interview, data collectors transcribed the responses onto hard copy forms for backup and verification.

As part of the larger project, a team of 12 trained midwife data collectors conducted the interviews under the supervision of three supervisors, each responsible for four data collectors (19). All data collectors and supervisors underwent a comprehensive nine-day training program covering data collection tools, ethical considerations, and procedures for conducting the two-week follow-up. Data submitted through the KoboCollect app were regularly checked for quality, completeness, and consistency, and the hard-copy forms were reviewed at the end of the data-collection period to ensure consistency and completeness.

## Measurement

*The level of PC-CAC*: Measured using the PC-CAC tool, which assesses the care provision in terms of informed decision-making, respect for individuals’ dignity, autonomy, privacy, and responsiveness to their needs and preferences (19, 20). The level of PC-CAC was categorized as low (below the 25th percentile), medium (25^th^-75th percentile), and high (above the 75^th^ percentile) (19–21). In our data, the cumulative PC-CAC score had a 25th percentile value of 53 and a 75th percentile value of 73 (19).

*Post-abortion contraceptive counseling*: Contraceptive counseling is one of the core aspects in CAC. In addition to the components used to assess the person-centeredness of CAC, we summarized counseling by asking women whether they had received contraceptive counseling following abortion care and before discharge, with responses recorded as “yes” or “no” (22, 23).

*Post-abortion contraceptive uptake*: Refers to a woman’s initiation and acceptance of a modern contraceptive method after completing an abortion procedure (24). Women were asked whether they had received a post-abortion contraceptive method following abortion care and before discharge, with responses recorded as “yes” or “no.” Women who reported receiving a contraceptive method before discharge were not followed up further, whereas those who reported not receiving a method were enrolled in a two-week follow-up to assess subsequent contraceptive uptake. Those who responded “yes” were subsequently asked to specify the type of contraceptive method they had received (23, 24).

## Data analysis

This study analyzed data from the same cohort established to assess the level of PC-CAC and its associated factors. Although it used data from the same cohort, the present analysis addressed a different objective by assessing the magnitude and associated factors of post-abortion contraceptive uptake.

Descriptive statistics, such as frequencies and percentages, were used to summarize the data, and the results were presented in tables. To identify factors associated with post-abortion contraceptive uptake, a binary logistic regression model was employed. Initially, bivariable logistic regression was conducted to identify variables with *p*-values < 0.25, which were then included in the multivariable logistic regression model.

Adjusted odds ratios (AORs) with 95% confidence intervals (25) were used to report statistically significant associations (*p* < 0.05) in the multivariable logistic regression model. The final multivariable analysis revealed that four key factors were significantly associated with post-abortion contraceptive uptake. The binary logistic regression model showed a good fit, as indicated by the Pearson chi-squared goodness-of-fit test, with a high *p*-value (Prob > chi^2^ = 0.1392), which is greater than the significance level of 0.05.

## Result

### Sociodemographic characteristics of the participants

A total of 1,642 participants were included in the analysis of post-abortion contraceptive uptake. All participants were assessed for contraceptive uptake before discharge. Of these, 438 participants had already received a post-abortion contraceptive method before discharge and therefore did not require further follow-up to assess contraceptive initiation. The remaining 1,204 participants who had not received a contraceptive method before discharge were enrolled in a two-week follow-up to assess subsequent contraceptive initiation. There was no loss to follow-up among these 1,204 participants, and all completed the two-week follow-up assessment. Among the respondents, the majority were aged 20–29 years (54%) and married (79%). Most were housewives (46.7%) and lived in urban areas (71%). About one-third (33.7%) had completed only primary education, and 42.5% had 1–2 live births. Nearly one-third (29.3%) were internally displaced. Further details on the participants’ sociodemographic characteristics are available in the first study (19).

### Reproductive and abortion care characteristics of study participants

Among the 1,642 participants, nearly two-thirds (65.9%) presented with incomplete abortions, while the remaining sought induced abortion services. A total of 8.3% reported that the pregnancy resulted from rape. Most abortions (70.8%) occurred during the first trimester, and 10.4% of women reported abortion-related complications at admission. Overall, 28.8% of participants had a history of spontaneous abortion, and 14.1% reported a prior induced abortion.

Regarding service delivery, one-third of abortion services were provided at referral hospitals, while the rest were delivered at general hospitals (28%), primary hospitals (23.1%), and health centers (18.6%). Midwives provided 62.9% of CAC services. Regarding the level of PC-CAC, approximately one-quarter of participants reported experiencing a low level of PC-CAC (Table 1).

**Table 1:** Reproductive and abortion service-related characteristics of the participants (*n* = 1,642).

Variable	*N*	(%)
Type of abortion at admission		
Induced abortion	560	34.1
Incomplete abortion	1,082	65.9
Previous history of spontaneous abortion		
No	1,170	71.2
Yes	472	28.8
Previous history of induced abortion		
No	1,410	85.9
Yes	232	14.1
Level of health facility		
Health center	306	18.6
Primary hospital	380	23.1
General hospital	459	28
Referral hospital	497	30.3
CAC provider		
Midwife	1,033	62.9
Physician	361	22
Others (Nurse, Health officer, IESO)	248	15.1
Abortion related complications at admission		
No	1,472	89.6
Yes	170	10.4
Perceived level of PC-CAC		
Low	425	25.9
Medium	829	50.5
High	388	23.6

### Post- abortion contraceptive counseling and uptake in the humanitarian setting

Nearly two-thirds of participants (62%) received post-abortion contraceptive counseling. Among those who received counseling, 41.5% received a post-abortion contraceptive method, compared with only 5.3% of those who did not receive counseling. Overall, post-abortion contraceptive uptake was 27.8%. Among those who received a post-abortion contraceptive method, 96% received the method before discharge, while 4% received the contraceptive method during the two-week follow-up period, all of whom obtained the method from other nearby health facilities.

Of the women who received post-abortion contraceptives, the majority (58.1%) chose injectable methods, followed by Implanon (28.1%) and oral contraceptive pills (11%).

Among women who received post-abortion contraceptive counselling, the majority presented with incomplete abortion (59.6%), while 40.4% were admitted for induced abortion. Over half of counselled women (52.3%) received a medium level of PC-CAC, and nearly one-third (29.3%) received a high level of PC-CAC. In contrast, only 18.4% of counselled women received a low level of PC-CAC.

Regarding facility type, among counselled women, the largest proportion (30.1%) received the care at the referral hospitals, followed by general hospitals (29.1%) and health centres (21.5%). Primary hospitals contributed the smallest share of counselling (19.3%). Most counselled women were attended by midwives (52.8%), followed by physicians (27.6%) and other providers (Nurse, Health officer, IESO) (19.6%).

Among women who received post-abortion contraceptives, 54.2% had presented with incomplete abortion, while 45.8% were admitted for induced abortion. The highest proportion (44.3%) of users received a medium level of PC-CAC, followed by (40.1%) high level of PC-CAC and (15.6%) low level of PC-CAC.

In terms of facility type, contraceptive uptake was highest in referral hospitals (31.1%), followed by health centers (27%) and general hospitals (24.8%), while primary hospitals accounted for the lowest proportion of uptake (17.1%). Most women who received contraceptives were attended by midwives (52.9%), with physicians (25%) and other providers (22.1%) (Table 2).

**Table 2:** Contraceptive counseling and uptake in relation to the abortion type, CAC quality, provider, and facility type (*n* = 1,642).

Characteristics	Contraceptive counseling *N* (%)		Contraceptive uptake *N* (%)	
	Yes	No	Yes	No
Type of abortion at admission				
Induced abortion	412 (40.4)	148 (23.8)	209 (45.8)	351 (29.6)
Incomplete abortion	608 (59.6)	474 (76.2)	247 (54.2)	835 (70.4)
Level of PC-CAC				
Low	188 (18.4)	237 (38.1)	71 (15.6)	354 (29.8)
Medium	533 (52.3)	296 (47.6)	202 (44.3)	627 (52.9)
High	299 (29.3)	89 (14.3)	183 (40.1)	205 (17.3)
Facility type				
Health center	219 (21.5)	87 (14)	123 (27)	183 (15.4)
Primary hospital	197 (19.3)	183 (29.4)	78 (17.1)	302 (25.5)
General hospital	297 (29.1)	162 (26.1)	113 (24.8)	346 (29.2)
Referral hospital	307 (30.1)	190 (30.5)	142 (31.1)	355 (29.9)
CAC provider				
Midwife	539 (52.8)	494 (79.4)	241 (52.9)	792 (66.8)
Physician	281(27.6)	80 (12.9)	114 (25)	247 (20.8)
Others (Nurse, Health officer, IESO)	200 (19.6)	48 (7.7)	101 (22.1)	147 (12.4)

### Factors associated with the post-abortion contraceptive uptake in the humanitarian setting

Based on the predefined criterion of *p* < 0.25, 10 variables were selected from the bivariable analysis as candidates for inclusion in the multivariable analysis: residence, marital status, educational status, previous history of spontaneous abortion, previous history of induced abortion, type of abortion, abortion procedure, health facility type, main CAC provider, and level of PC-CAC provision. In the multivariable logistic regression analysis, four factors were statistically significantly associated with post-abortion contraceptive uptake (*p*-value < 0.05): being single, having a previous history of spontaneous abortion, a previous history of induced abortion, and a high level of PC-CAC provision.

The odds of post-abortion contraceptive uptake were significantly higher among women with single marital status [AOR: 1.96; 95% CI: 1.39–2.75], a previous history of induced abortion [AOR: 2.00; 95% CI: 1.42–2.83], and those who received a high level of PC-CAC services [AOR: 3.39; 95% CI: 2.39–4.82]. In contrast, the odds were significantly lower among women and girls with a previous history of spontaneous abortion [AOR: 0.73; 95% CI: 0.55–0.97] (Table 3).

**Table 3:** Factors associated with the post-abortion contraceptive uptake in the humanitarian setting (*n* = 1,642).

Variables	Post-abortion contraceptive uptake *N* (%)		COR (95% CI)	AOR (95%CI)	*p*-value
	Yes	No			
Residence					
Urban	349 (29.8)	822 (70.2)	1.44 (1.13–1.85)	1.13(0.85–1.51)	0.407
Rural	107 (22.7)	364 (77.3)	1	1	
Marital status					
Married	299 (23)	998 (77)	1	1	
Single	122 (48.8)	128 (51.2)	3.18 (2.41–4.21)	1.96 (1.39–2.75) *	<0.001
Divorced	28 (35.4)	51 (64.6)	1.83 (1.14–2.96)	1.16 (0.69–1.97)	0.570
Widowed	7 (43.7)	9 (56.3)	2.59 (0.96–7.03)	1.44 (0.48–4.34)	0.513
Educational status					
Cannot read or write	73 (21.7)	263 (78.3)	1	1	
Can read and write (without formal education)	24 (28.6)	60 (71.4)	1.44(0.84–2.47)	1.38 (0.77–2.47)	0.279
Primary (1-8)	163 (29.5)	390 (70.5)	1.51 (1.09–2.07)	1.21 (0.85–1.72)	0.291
Secondary (9-12)	132 (30.4)	302 (69.6)	1.57 (1.13–2.19)	1.03 (0.69–1.51)	0.894
College and above	64 (27.2)	171 (72.8)	1.35 (0.92–1.99)	0.88 (0.5–1.37)	0.559
Previous history of spontaneous abortion					
Yes	98 (20.8)	374 (79.2)	0.59 (0.46–0.77)	0.73 (0.55–0.97) *	0.029
No	358 (30.6)	812 (69.4)	1	1	
Previous history of induced abortion					
Yes	123 (53)	109 (47)	3.65 (2.74–4.86)	2.00 (1.42–2.83) *	<0.001
No	333 (23.6)	1,077(76.4)	1	1	
Type of abortion at admission					
Induced abortion	209 (37.3)	351 (62.7)	2.01 (1.61–2.51)	1.01 (0.74–1.36)	0.970
Incomplete abortion	247 (22.8)	835 (77.2)	1	1	
Abortion procedure					
Medical abortion (MA)	236 (35.3)	433 (64.7)	1.88 (1.47, 2.40)	1.23 (0.90–1.66)	0.189
Manual vacuum aspiration (MVA)	140 (22.5)	483 (77.5)	1	1	
Both MA and MVA	80 (22.9)	270 (77.1)	1.02 (0.75–1.39)	0.89 (0.63–1.27)	0.540
Health facility type					
Health center	123 (40.2)	183 (59.8)	1.68 (1.24–2.27)	1.12 (0.79–1.59)	0.510
Primary hospital	78 (20.5)	302 (79.5)	0.65 (0.47–0.89)	0.91 (0.63–1.32)	0.632
General hospital	113 (24.6)	346 (75.4)	0.82 (0.61–1.09)	0.94 (0.67–1.31)	0.708
Referral hospital	142 (28.6)	355 (71.4)	1	1	
Main CAC provider					
Midwife	241 (23.3)	792 (76.7)	0.44 (0.33–0.59)	0.83 (0.59–1.17)	0.282
Physician	114 (31.6)	247 (68.4)	0.67 (0.48–0.94)	0.99(0.67–1.49)	0.989
Others (Nurse, Health officer, IESO)	101 (40.7)	147 (59.3)	1	1	
Level of PC-CAC provision					
Low	62 (14.6)	363 (85.4)	1	1	
Medium	211 (25.5)	618 (74.5)	1.99 (1.46–2.73)	1.31 (0.95–1.81)	0.095
High	183 (47.2)	205 (52.8)	5.23 (3.74–7.31)	3.39 (2.39–4.82) *	<0.001

*Variables showed a significant association with *p* ≤ 0.05. The value “1” indicates the reference category.

## Discussion

Our study found that only one-quarter of women and girls in the humanitarian setting were using post-abortion contraception. Post-abortion contraceptive uptake was positively associated with being single, having a prior history of induced abortion, and receiving a high level of PC-CAC. In contrast, a history of spontaneous abortion was negatively associated with contraceptive uptake.

Contraceptive uptake in this study was higher than that reported in Nigeria but notably lower than that reported in other humanitarian settings, such as the CAR (8). This difference may be largely attributable to variations in the provision of contraceptive counselling, a well-established determinant that enhances informed decision-making and contributes to higher contraceptive uptake (26). In the Nigerian setting, counselling before discharge was more limited than in our study, reflecting comparatively stronger counselling provision within post-abortion care services in our context (8). Differences in service integration and accessibility likely account for the discrepancy in findings with the CAR (27). In the CAR setting, contraceptive services were fully integrated into PAC and delivered under a unified management structure, with systematic pre-discharge counselling coverage (8, 26). In contrast, in our humanitarian context, contraceptive services were poorly integrated with PAC, sometimes managed by separate departments, and often constrained by the unavailability of contraceptive methods (28, 29). These service delivery barriers likely limited immediate access and reduced post-abortion contraceptive uptake at the point of care (27).

With respect to the factors influencing post-abortion contraceptive uptake, single women were significantly more likely to use post-abortion contraceptives compared to those who were married. This finding aligns with other studies (10, 30, 31). The fear of economic dependency and the strong cultural stigma surrounding unmarried pregnancy, which is more common in a humanitarian context where daily life is precarious and resources are scarce, may lead women to consider post-abortion contraceptives to prevent future unintended pregnancies (32, 33). Conversely, some studies also showed that single women are less likely to use a post abortion contraceptive (34–39). This is because young, unmarried women may have less autonomy in making reproductive choices, with decisions strongly influenced by family or social networks (40).

This study also revealed that women who had a previous history of induced abortion were more likely to have post-abortion contraceptive uptake in the humanitarian context. Other studies also support this finding (35, 36, 41). This may be due to the experience of an unintended pregnancy and undergoing an induced abortion, particularly in humanitarian settings, where stressful and stigmatizing conditions motivate women to avoid repeating such experiences by choosing to use contraception after an abortion (42). Conversely, women with a history of spontaneous abortion were less likely to use contraceptives after abortion. This finding is consistent with other studies (43, 44) and may be because women do not prefer to use contraception, particularly those who desire a pregnancy and typically plan to have a child. Therefore, they may seek to regain fertility as quickly as possible to conceive again (43).

With respect to quality of care in the PC-CAC approach, participants who received a high level of PC-CAC were more likely to use a post abortion contraceptive than women who received lower PC-CAC services. This finding is consistent with studies done in Nigeria and the CAR (8). The quality of abortion care provision is crucial in humanitarian or fragile settings where access to information and services is often compromised; creating an individualized and supportive environment may contribute to positive care experiences and improve post-abortion contraceptive uptake (3, 8). In humanitarian settings, insecurity, population displacement, workforce shortages, frequent contraceptive stock-outs, and fragmented service delivery collectively shape the post-abortion care experience (45, 46). These systemic constraints create substantial barriers to the timely uptake of effective contraception by limiting access to quality counselling, reducing method availability, and increasing reliance on external referrals, undermining women’s ability to make informed and autonomous reproductive choices (3).

Although provider type was expected to influence uptake, no statistically significant association was found between provider cadre and contraceptive uptake. Descriptive findings suggest that multiple provider groups play an important role in post-abortion contraceptive counseling and uptake, with the highest proportion of uptake provided by midwives. Therefore, these differences should be interpreted cautiously, as they may reflect variations in case mix, service delivery context, and scope of practice rather than differences in provider performance. In this study, the higher contraceptive uptake observed among women attended by midwives may reflect the fact that midwives provided most CAC services. However, existing evidence also highlights the important role of midwives in promoting positive experiences with post-abortion contraceptive counseling and improving contraceptive uptake among women receiving post-abortion care (47).

The descriptive finding of the highest proportion of post-abortion contraceptive uptake was among women who received CAC at the referral hospital, followed by health centers. This might be explained by the fact that most CACs were provided at referral hospitals. Previous evidence indicates that referral hospitals have higher uptake of long-acting contraceptive methods because they are better equipped with the clinical expertise, equipment, and commodities required for long-acting contraceptive provision (15, 48). In contrast, although primary-level facilities such as health centers may have more limited capacity to provide these methods, they often achieve higher overall contraceptive uptake. This may be because health centers typically manage less complex cases, allowing providers to provide more personalized and client-centered counseling, supporting higher uptake (11). Furthermore, the closer proximity of health centers to communities may also foster trust, continuity of care, and willingness to initiate contraception at the point of service (48, 49).

Overall, this study underscores how crisis-affected contexts continue to undermine women’s reproductive autonomy and the delivery of integrated, quality abortion care. From a reproductive justice perspective, the observed lower uptake should not be interpreted as individual non-compliance or lack of demand, but rather as a reflection of structural and contextual constraints, including service fragmentation, limited contraceptive choice, provider capacity gaps, insecurity, and disrupted continuity of care that shape women’s ability to make informed and voluntary reproductive decisions (50). This highlights that, while contraceptive uptake is a key indicator of quality abortion care, the extent to which services uphold dignity, self-determination, and freedom from coercion is crucial to ensuring the principles of reproductive and contraceptive justice (51).

Our findings highlight an urgent need for targeted policy and practice interventions to enhance post-abortion contraceptive uptake in Ethiopia’s humanitarian settings. Aligning with the Health Sector Transformation Plan (HSTP II), policies should prioritize the integrated, point-of-care delivery of abortion and contraceptive services, ensuring that abortion services, counselling, and method provision occur within a single encounter to prevent missed opportunities and minimize delays (52, 53). Strengthening integration not only reduces the risk of repeat unintended pregnancies, which carry significant health, social, and economic burdens for women and the health system, but also reinforces continuity of care and informed decision-making (54). Globally, these findings reveal persistent gaps in achieving SDG 3.7 on universal access to sexual and reproductive health services and SDG 5.6 on reproductive autonomy (55, 56). The substantially lower uptake observed in humanitarian contexts underscores that conflict and displacement-affected women and girls remain disproportionately underserved, challenging Ethiopia’s commitments to Universal Health Coverage and the SDG principle of leaving no one behind (57, 58). Addressing these gaps requires sustained investment in service integration, supply chain reliability, workforce capacity, and person-centered approaches, ensuring that national strategies translate into equitable reproductive health outcomes even in crisis-affected areas (56, 59).

This study has several strengths. It included a large sample of women receiving comprehensive abortion care across multiple levels of the public health system in a conflict-affected setting, where evidence on post-abortion contraceptive counselling and uptake remains limited. The extended data-collection period captured service delivery under changing humanitarian conditions and may increase the relevance of the findings beyond a single point in time. However, the facility-based design limits generalizability to women who accessed participating public facilities and does not represent those receiving care elsewhere or unable to obtain care. The cross-sectional design also precludes causal inference and limits conclusions about the temporal relationships between associated factors and contraceptive uptake. Social desirability and recall bias cannot be excluded, particularly for sensitive reproductive histories and experiences of care. These risks were reduced through private interviews, neutral and non-leading questions, and rigorous interviewer training. Finally, contraceptive uptake was measured both before discharge and at the two-week follow-up. Differences in timing may have affected the cumulative uptake estimate; therefore, immediate and follow-up uptake were reported separately with clear denominators.

Future research should prioritize comparative studies between humanitarian and non-humanitarian settings to better understand how context mediates reproductive choice, counselling quality, and decision-making autonomy. Such comparative inquiry can help disentangle context-specific barriers from broader systemic challenges, generate evidence on how care environments influence post-abortion contraceptive decision-making and uptake, and inform the development of context-responsive interventions that promote reproductive and contraceptive justice.

## Conclusion

Post-abortion contraceptive uptake in humanitarian settings remains low; however, from a reproductive justice perspective, this gap should be understood primarily because of structural and contextual barriers rather than a lack of individual demand. Service fragmentation, limited contraceptive choice, provider capacity constraints, insecurity, and disruptions in continuity of care collectively restrict women’s ability to make informed and voluntary reproductive decisions. Addressing these challenges requires strengthening the quality of abortion care in ways that uphold dignity, autonomy, and freedom from coercion. Post-abortion contraceptive care in humanitarian settings should prioritize informed and voluntary decision-making for all women, with sensitivity to diverse reproductive histories, including experiences of spontaneous or induced abortion. Strengthening integrated, person-centered counselling and method provision at the point of care is essential to ensuring that women’s reproductive health needs are met, even in the most challenging contexts.

## Data Availability

The raw data supporting the conclusions of this article will be made available by the authors, without undue reservation.
